# Tissue discrimination in head and neck cancer using image fusion of IR and optical microscopy†

**DOI:** 10.1039/d3an00692a

**Published:** 2023-07-27

**Authors:** Safaa Al Jedani, Caroline I. Smith, James Ingham, Conor A. Whitley, Barnaby G. Ellis, Asterios Triantafyllou, Philip J. Gunning, Peter Gardner, Janet M. Risk, Richard J. Shaw, Peter Weightman, Steve D. Barrett

**Affiliations:** a Department of Physics, University of Liverpool L69 7ZE UK peterw@liverpool.ac.uk; b Department of Physics, University of Jeddah Saudi Arabia; c Department of Cellular Pathology, Liverpool Clinical Laboratories, University of Liverpool Liverpool L7 8YE UK; d Liverpool Head and Neck Centre, Department of Molecular and Clinical Cancer Medicine, University of Liverpool L7 8TX UK; e Manchester Institute of Biotechnology, University of Manchester 131 Princess Street Manchester M1 7DN UK; f Head and Neck Surgery, Liverpool University Foundation NHS Trust, Aintree Hospital Liverpool L9 7AL UK

## Abstract

A regression-based fusion algorithm has been used to merge hyperspectral Fourier transform infrared (FTIR) data with an H&E image of oral squamous cell carcinoma metastases in cervical lymphoid nodal tissue. This provides insight into the success of the ratio of FTIR absorbances at 1252 cm^−1^ and 1285 cm^−1^ in discriminating between these tissue types. The success is due to absorbances at these two wavenumbers being dominated by contributions from DNA and collagen, respectively. A pixel-by-pixel fit of the fused spectra to the FTIR spectra of collagen, DNA and cytokeratin reveals the contributions of these molecules to the tissue at high spatial resolution.

## Abstract 2

Fusion of FTIR with H&E images reveals the contributions of collagen, DNA and cytokeratin in metastatic/lymphoid tissue at high spatial resolution.

## Graphical abstract

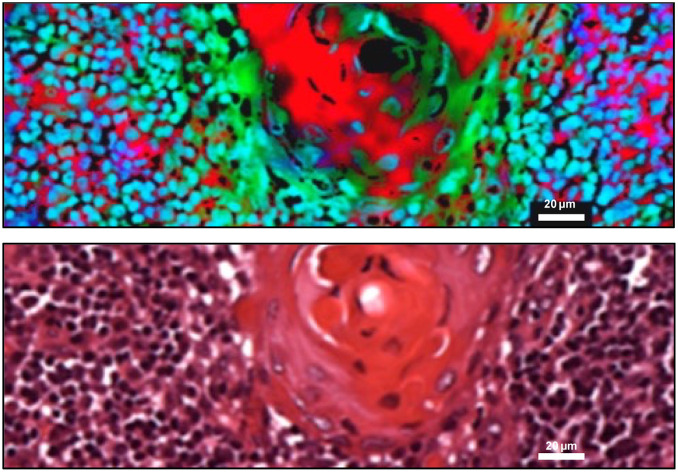

## Introduction

Histology and histochemistry/immunohistochemistry are standard and time-honoured technologies in studying the morphology and chemical composition in relation to location of cells and tissues, and are pivotal in reaching pathological diagnosis. In the case of cancer, except for establishing diagnosis and pathological grading, they also yield valuable information on prognosticators.^1–4^ Nonetheless development of alternative, complementary technologies is desirable. The latter include label-free infrared (IR) imaging, which provides spectral resolution sufficient to reveal some basic information on chemical structure and is showing some promise as a diagnostic tool.^5–13^ However, IR imaging is limited by low spatial resolution resulting in pixel sizes that cannot resolve details in cells and tissues,^9^ and it would be beneficial to combine the spectral and spatial information from IR and histology in fused images.

Earlier attempts to fuse images used Raman, rather than histology, and Fourier transform IR (FTIR) hyperspectral images using a multivariate curve resolution alternating least squares (MCR-ALS) technique.^14^ Later studies adopted an image super-resolution approach to enhance the IR image resolution by a training generative adversarial network (GAN) model using routine histological haematoxylin and eosin (H&E) stained sections of breast tumour biopsies as a high-resolution image source.^15^ The approach allowed reconstruction of the IR low-resolution image based on the H&E high-resolution image. In addition, an unsupervised curvelet-based image fusion method effected fusing images of breast and ovarian tumour biopsies acquired from IR and dark-field microscopy.^16^ In general, fusion algorithms differ in their performance and computational load. The most important difference between these fusion algorithms is in calculating the weight (*i.e.*, balance the input of high-resolution and low-resolution image) which is established by solving an optimisation problem.^17^

Recently a regression-based fusion algorithm^18^ was used to merge paired, co-registered H&E and IR images of three specimens of oral squamous cell carcinoma (OSCC) metastases in cervical lymph nodes. FTIR spectral images of similar specimens have also been analysed using a machine learning algorithm (MLA),^19^ which revealed that a single “metric”, the ratio of image intensities at 1252 cm^−1^ and 1285 cm^−1^, was able to discriminate between metastasis and lymphoid tissue with sensitivities, specificities and precision of 98.8 ± 0.1%, 99.89 ± 0.01% and 99.78 ± 0.02% respectively. Aperture scanning near-field optical microscopy (SNOM) images of the tissue obtained at a number of key wavenumbers showed variations in the chemical composition of the tissues, but identification of corresponding cellular elements was problematic. In an attempt to rectify this, the fused images presented here allow comparison with the results of the previous MLA and SNOM studies providing further insight into the chemical composition of OSCC metastasis.

## Experimental

### Sample preparation

Archival, formalin-fixed, paraffin-embedded (FFPE) tissue blocks from cervical lymph node metastases were obtained from a single patient with OSCC following informed consent. All experiments were performed under the sponsorship of the University of Liverpool and with the ethical approval of the Northwest Liverpool Central Research Ethics Committee (REC number EC 47.01). A region of interest (ROI) containing both metastatic OSCC and surrounding lymphoid tissue was identified by light microscopy on sections routinely prepared and stained with H&E. Cores of 1 mm diameter containing the ROI were removed from FFPE blocks using a Beecher MTA-1 tissue microarrayer for remounting in paraffin. Adjacent 5 μm sections of this ‘daughter’ block were cut and mounted onto calcium fluoride (CaF_2_) disks for FTIR experiments and onto charged glass slides for H&E staining. Although multiple cores were taken, only one core was deemed appropriate for study (as judged from the H&E section). Sections for FTIR were retained in the paraffin wax to prevent alterations in hydration and structure of the tissue. H&E stained sections were scanned using the Aperio CS2 scanner (Leica Biosystems, Milton Keynes, UK).

### Data acquisition

IR data were collected using an Agilent Cary 620 IR microscope connected to an Agilent Cary 670-IR spectrometer (Agilent, Stockport, UK) as described previously.^20,21^ Briefly, collection was from 3800 cm^−1^–990 cm^−1^ using 64 co-added scans (128 for background) at a spectral resolution of 4 cm^−1^ with an effective image pixel size of 5.5 μm, and resulted in a FTIR datacube with both spatial and spectral dimensions. Poor-quality spectra, with amide I absorbance less than 0.1 or greater than 2.0, were removed from the dataset. Spectral regions dominated by paraffin contributions (1350 cm^−1^–1500 cm^−1^ and 2835 cm^−1^–3000 cm^−1^) were omitted from the analysis, and spectra were truncated to the fingerprint region (900 cm^−1^–1800 cm^−1^). A rubber-band baseline correction^22^ was applied to each spectrum in the truncated dataset, followed by vector normalisation. Embedding of the tissues in paraffin reduced scattering artefacts—hence Mie scattering correction^10^ was not strictly necessary for the bulk tissue due to the refractive index matching between the tissues and paraffin, but one iteration was applied to all spectra to account for tissue edge effects.

The data were pre-processed in the following order: rubber-band correction, noise reduction, scattering correction, quality test, truncation of regions most affected by paraffin (1350 cm^−1^–1500 cm^−1^ and 2835 cm^−1^–3000 cm^−1^) and lastly, vector normalisation.

### Fusion model and quality metrics

Pixel-level image fusion was applied to the FTIR datacube as previously described.^18^ Briefly, manual image registration was used to correct any misalignment between H&E and FTIR images (Adobe Photoshop 2021 v22). FTIR images were upsampled using bicubic interpolation to the same spatial *x*–*y* scale as the H&E image (0.25 μm). The H&E image was converted into greyscale and then standardised (scaled) by subtracting the mean and dividing by the standard deviation.^18^ A pixel-level fusion algorithm based on a linear regression model was used to combine high-resolution and low-resolution images.^23^ Spectral distortion was minimised by equalising the fused image with the FTIR image. Lastly, quality metrics were applied to the images and the spectra to ensure that neither showed significant distortions. The structural similarity index measure (SSIM)^24^ (Python scikit-image processing library^25^) was used to assess the extent of spatial correlation between the fused and the H&E images. An SSIM score of 1 indicates that the two images are identical; a lower value indicates less structural similarity. Spectral distortion was quantified using a spectral angle mapper (SAM) that measures spectral similarity by calculating the angular distortion between spectra treated as vectors in multidimensional space^26^ and was implemented using the MATLAB Image Processing Toolbox^27^ using the FTIR images as the reference. The SAM score for identical spectra is zero, while a higher value indicates that the spectral data is distorted.

Spectral cluster analysis was implemented using the MATLAB Statistics and Machine Learning Toolbox.^28^

### Molecular spectral fits

Each pixel (spectrum) of the fused images was fitted to the IR spectra of three molecules, chosen for their biological relevance for the particular tissues.^14,29^ A least-squares fit (LSF) implemented in Python produced the contributions of these molecules which were then assigned to the three (red-green-blue) colours of the corresponding pixels of an RGB image.

## Results

**Fig. 1:**
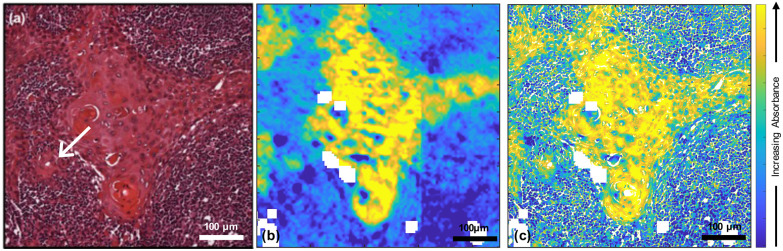
(a) H&E-stained cervical lymph nodal metastasis of OSCC with an arrow indicating the region discussed in the text; (b) FTIR image at 1650 cm^−1^ taken from the adjacent section; (c) fused image of (a) and (b). The images (b) and (c) are shown over the range of the 95^th^ percentiles.

Fused images were created for all the wavenumbers in the fingerprint region producing high resolution images with infrared information (Fig. 1).

In order to evaluate the quality of the fused images of this core, both SSIM and SAM were used along with peak signal to noise ratio (PSNR) and mean squared error (MSE) (ESI Fig. 1†). The SSIM scores for all the fused images ranged from ∼0.7 to ∼0.9; due to the importance of maintaining the chemical information from the FTIR image, the contrast components between optical H&E and fused images were not expected to be identical. Scores for all the fused images are higher than those for the FTIR images, which ranged from ∼0.1 to ∼0. 2. The SAM scores ranged from ∼0.001 to ∼0.2.

**Fig. 2:**
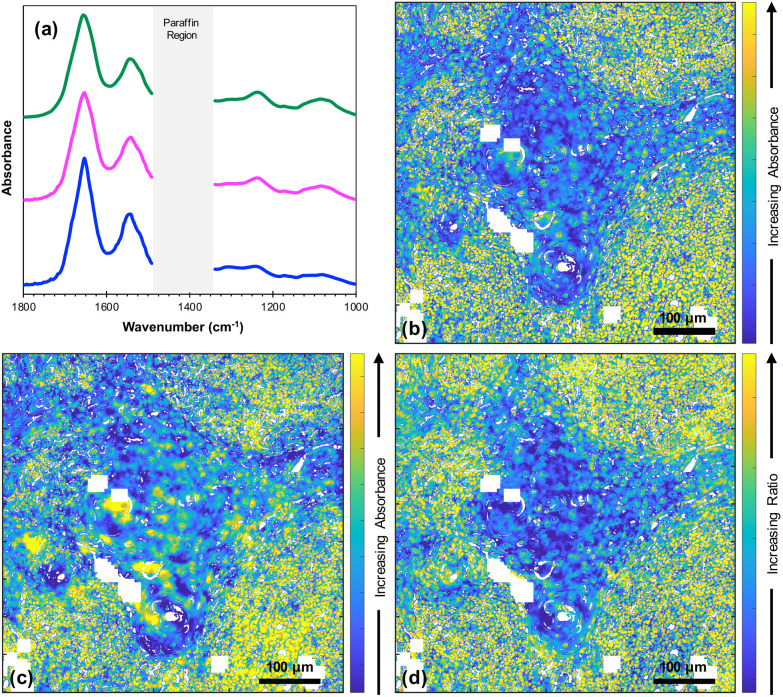
(a) Average FTIR spectra taken from lymphoid cells (green line), tumour (pink line) and highly keratinised tumour areas (blue line) (offset for clarity); (b) fused image at 1252 cm^−1^; (c) fused image at 1285 cm^−1^; (d) fused ratio image at 1252/1285. The images (b), (c) and (d) are shown over the range of the 95^th^ percentiles. The white pixels denote poor-quality spectra as defined in Data Acquisition.

Fig. 2(a) shows the average FTIR spectra of lymphoid cells (59 230 spectra), tumour (114 970 spectra) and highly keratinised tumour areas (10 265 spectra) that were obtained from a *k*-means cluster analysis of the datacube. It is clear that all these spectra are very similar. However, images at two specific wavenumbers^14^ and the ratio of absorbances at those two wavenumbers showed clear contrast between the biological moieties [Fig. 2(b), (c) and (d)].

**Fig. 3:**
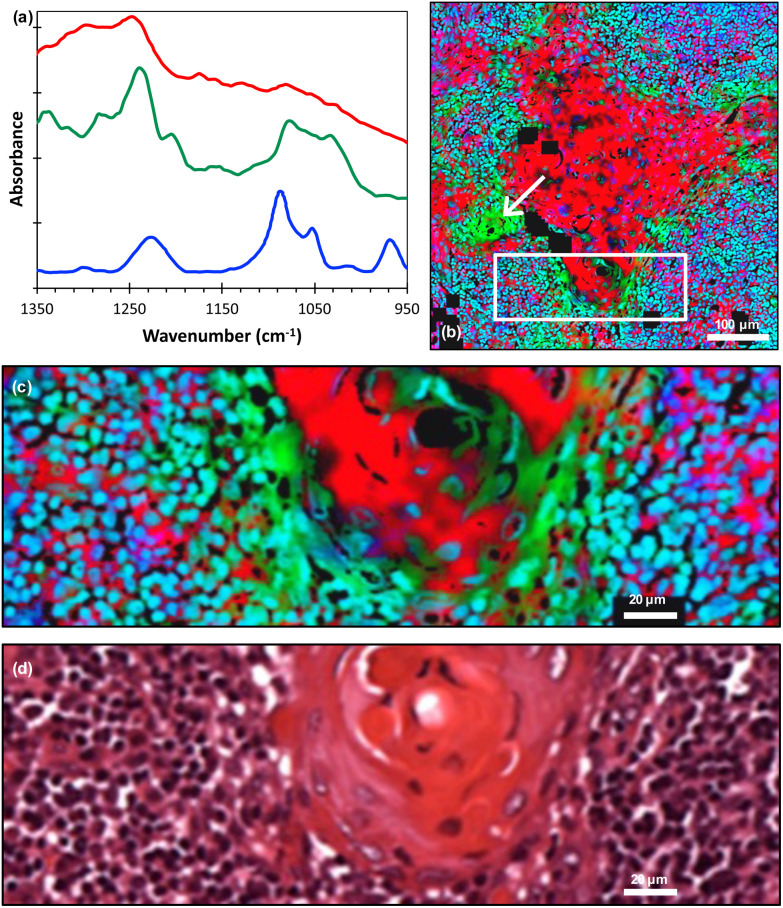
(a) FTIR spectra of cytokeratin (red line), collagen (green line) and ds-DNA (blue line) adapted from ref. 30–32 (offset for clarity); (b) least-squares fit of the FTIR spectra of these molecules displayed as an RGB composite image (red = cytokeratin, green = collagen and blue = ds-DNA) with an arrow indicating the region discussed in the text; (c) magnified image taken from the white rectangle shown in (b); (d) corresponding H&E image. The individual RGB channels of image (c) are shown in ESI Fig. 2.†

The literature and our own previous work suggest that three key molecules, DNA and molecules from the collagen and cytokeratin families, are major contributors to OSCC and supporting stroma tissue composite.^14,29^ Instead of producing fused images at single wavenumbers that have been assigned to known vibrational modes in particular moieties, we considered the spectra of the three key molecules^30–32^ [Fig. 3(a)] and performed pixel-by-pixel least-squares fits to the fused FTIR spectra. The goodness-of-fit parameter *χ*^2^ was typically in the range 2 to 5. The contribution of each molecule was then displayed as a composite RGB image, with cytokeratin shown in the red channel, collagen in the green channel and double stranded (ds)-DNA in the blue channel [Fig. 3(b)] and any pixel that did not produce a good fit shown as black. A magnified image taken from the boundary of the metastasis/lymphoid regions [Fig. 3(c)] showed variations in the chemical composition of different topographical compartments of the microenvironment [Fig. 3(d)].

It should be noted that we lack absolute scales as ref. 30–32 did not quantify absorbance scales on which the molecular spectra were plotted. Hence, the RGB image gives the spatial distributions of these molecules but not their relative concentrations.

## Discussion

The fusion of optical and IR images has the potential to combine high resolution spatial and spectral information, respectively, within the same image.^11–13^ In the current study, the spatial resolution of fused images (<1 μm) is considerably greater than the original FTIR images (∼10 μm). Furthermore, the quality metrics, SSIM and SAM, indicated both good spatial correlation with the original H&E image and low spectral distortion compared with the original FTIR image, respectively. The findings suggest that the fusion procedure loses little information from either the optical or the FTIR image.

It is useful to compare the results of this study with a previous study of fused images of non-cancerous oral epithelium and underlying stroma.^18^ The two tissue types were clearly delineated in both the H&E and FTIR spectral images shown in Fig. 4 of ref. 18 and histopathologists have a good understanding of these tissue types. Consequently, the differences in contrast between the two tissue types at five different FTIR wavenumbers could be understood in terms of contributions from DNA and collagen. The analysis was supported by an FTIR image obtained at a wavenumber attributed to lipids. It is notable that the success in interpreting the spectral images of Fig. 4 of ref. 18 was obtained in the absence of knowledge of the relative intensities of the DNA and collagen FTIR spectra. This was only possible because of the clear delineation in the images of the two tissue types.

In the earlier study of lymphoid nodal tissue and metastatic OSCC, an MLA achieved excellent discrimination between the two tissue types in terms of the ratio of absorbances at 1252 cm^−1^ and 1285 cm^−1^. These two wavenumbers were attributed to contributions dominated by DNA and collagen, respectively.^19^ The fused images obtained in this current work are consistent with those of ref. 19 as shown in Fig. 2(d), which is in better agreement with the H&E image of Fig. 1(a) than the images obtained at the individual wavenumbers of 1252 cm^−1^ and 1285 cm^−1^. It is worth exploring why the ratio image is successful, and whether the attribution of this success solely to contributions from DNA and collagen is reasonable when it is known that a third moiety, cytokeratin, makes an important contribution to the tissue. Ignoring cytokeratin, and other moieties, for the moment we can write the ratio as:1
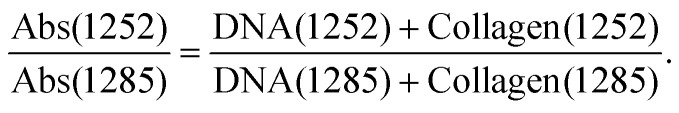


The fused ratio image has low values in the core of the tumour–cell aggregates, which tends to show higher differentiation in the form of large cells showing increased cytoplasm enriched with cytokeratins and lower nuclear-to-cytoplasmic ratios. The absorbance ratio is higher in the non-tumour regions, emphasising the nuclei of the lymphoid cells. This is expected as lymphocytes show little cytoplasm, which results in more closely packed nuclei in the lymphoid tissue compared with the less closely packed nuclei of the differentiated centres of tumour-cell aggregates.^19^ The success of eqn (1) in accounting for the distribution of DNA indicates that the numerator is dominated by the contribution from DNA and the denominator by the contribution from collagen. Thus, even in the absence of knowledge of the relative intensity of the DNA and collagen spectra, we can conclude that the collagen contributions at 1252 cm^−1^ and the DNA contributions at 1285 cm^−1^ are both relatively weak, which is consistent with the original attribution of these wavenumbers to DNA and collagen, respectively.

As previously noted, three molecular contributors that are important in OSCC and supported stroma composite are collagens, DNA and cytokeratins.^29,33^ In this microenvironment, cytokeratins are localised in the cytoplasm of tumour cells, collagens in the stroma and DNA in the nuclei of tumour cells and immune/inflammatory cells in the stroma. The localisation has been repeatedly confirmed by multiple histochemical and immunohistochemical investigations. Instead of producing fused images at individual wavenumbers that have been assigned to known vibrational modes in particular chemical moieties, we have included all three key molecules in the analysis of the fused FTIR images by performing a least-squares fit pixel-by-pixel to model the contributions of these molecules. This has the advantage of using all of the spectral information for each molecule and allowing sub-cellular observation of molecular contributions. Combinations of more than just these three molecules were tested by considering the addition of other molecules that might be present in the tissue (such as RNA, other proteins, glycogen) but none improved on the results discussed here.

On the larger spatial scale [Fig. 3(b)], the core of the tumour-cell aggregates appears mostly red, indicating a large cytokeratin component to the tissue, whereas the edge of the aggregates is green, indicating extracellular collagen deposits. Also, we note that the collagen was associated with keratin ‘pearls’, which is consistent with previous FTIR work.^29^ The largest region labelled as collagen [indicated by the arrows in Fig. 3(b) and 1(a)] appears in the H&E image to be a keratinised region, implying a possible misinterpretation or a change occurring between adjacent sections. A cytokeratin stain (ESI Fig. 3†) of an adjacent tissue section showed that this area is not keratinised. Hence, the least-squares fitting has identified the presence of a molecule in a region that is not readily apparent from the H&E alone. Thus, the FTIR data has provided information that would otherwise require staining of an additional tissue section.

At higher spatial resolution [Fig. 3(c)] it is apparent that these peripheral areas are not coloured uniformly but show overlap with other spectra, as indicated by changes in colour and intensity. The lymphoid cell nuclei are mostly shown as blue or cyan in colour, confirming the known overlap between the DNA (blue) and collagen (green) spectra.^31,32^ Accordingly, while areas known histologically to be rich in collagen can be delineated, the non-uniform and complex appearances at the periphery of the tumour-cell aggregates are attributable to nuclei of immune cells adjacent to the nuclei of tumour cells. The periphery of tumour-cell aggregates tends to show less differentiation in the form of smaller cells, showing little cytoplasm with decreased cytokeratins and higher nuclear-to-cytoplasmic ratios. This would result in more closely packed tumour nuclei therein.

These observations at high spatial resolution could not have been made without the fusion of the FTIR images with an H&E image. This has been demonstrated by applying the same pixel-by-pixel fit to the original (unfused) FTIR data as shown in ESI Fig. 4.†

## Conclusions

The image fusion procedure described here is capable of combining spectral and high spatial information with only minor loss of either and confirms that the 1252 cm^−1^/1285 cm^−1^ ratio is a good discriminant of metastatic OSCC within the lymphoid nodal tissue at high spatial resolution. The success of this ratio of absorbances in discriminating between the tissue types is due to the absorbances at the 1252 cm^−1^ and 1285 cm^−1^ being dominated by contributions from DNA and collagen, respectively. Other moieties will contribute to the spectral absorbance at these two wavenumbers, notably cytokeratin, but it is clear from this and previous work^14^ that it is the difference in the contributions from these two molecules that accounts for the success of this ratio of absorbances in discriminating between the two tissue types.

Fitting spectra of collagen, DNA and cytokeratin to the fusion of hyperspectral FTIR data and an H&E image gives excellent agreement with histopathological analysis and reveals the distributions of these molecules at high spatial resolution. This demonstrates the potential of image fusion to both identify and locate chemical moieties in a way that is not possible with either FTIR or H&E alone.

## Data availability

The data will be available *via* the University of Liverpool Data Catalogue.

## Author contributions

SAJ designed the experiment, wrote the algorithm used for the fusion of FTIR and H&E images prepared the figures, analysed the data, and prepared the first draft. CIS designed the experiment, prepared the figures, analysed the data, administrated the project and prepared the first draft. JI wrote the machine learning algorithm that identified the metric and helped with image processing. CAW and BGE collected the FTIR data. PJG prepared the tissue microarrays, sectioned and stained tissue and dewaxed samples for imaging. PG provided access to the FTIR microscope and supervised the FTIR experiments. SDB analysed the data, supervised the work, obtained the funding, administrated the project and prepared the first draft. AT designed the experiment, classified and annotated the stained samples. JMR designed the experiment, selected the tissue samples, analysed the data, supervised the work, obtained the funding, administrated the project and prepared the first draft. RJS supervised the work and obtained the funding. PW designed the experiment, analysed the data, supervised the work, obtained the funding, administrated the project and prepared the first draft. All authors were involved in a critical review and edit of the paper.

## Conflicts of interest

There are no conflicts to declare.

## Supplementary Material

AN-148-D3AN00692A-s001
