# Supplementary material for: Tissue discrimination in head and neck cancer using image fusion of IR and optical microscopy
Source: Analyst. 2023 Jul 27;148(17):4189–94. doi: 10.1039/d3an00692a (PMC10440831; doi:10.1039/d3an00692a)
Supplement: AN-148-D3AN00692A-s001 [file AN-148-D3AN00692A-s001.pdf]

Electronic Supplementary Material (ESI) for Analyst.
This journal is © The Royal Society of Chemistry 2023

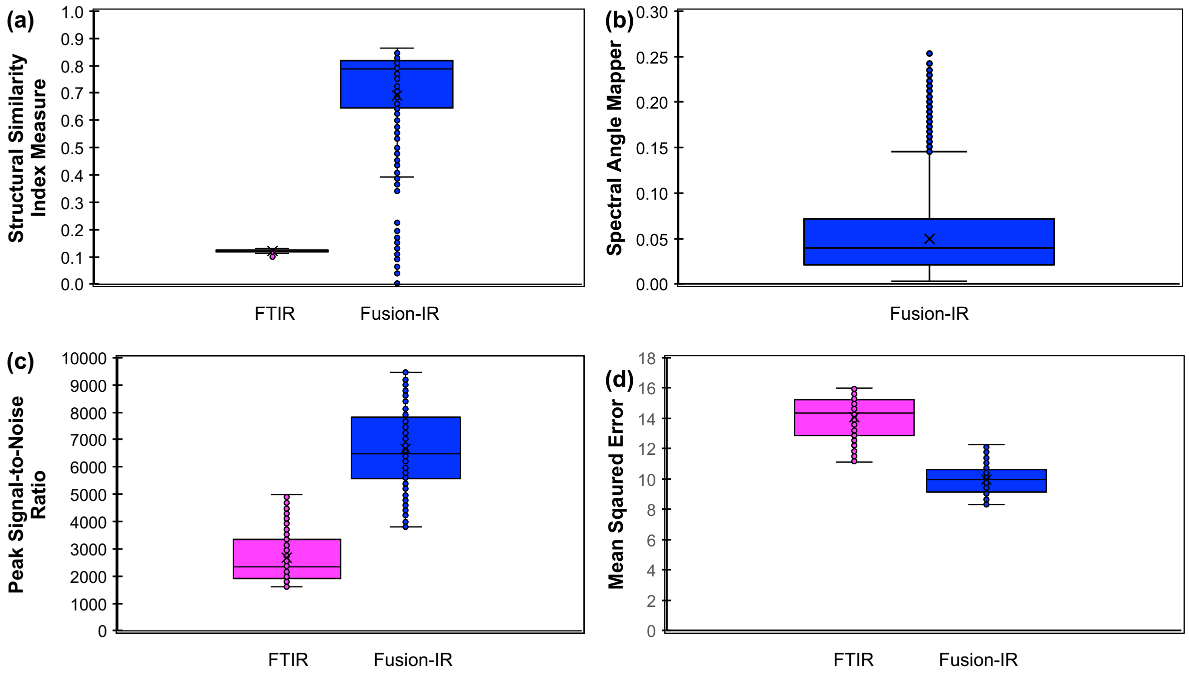

Fig. S1: Quality comparison of pre- and post-fusion images and spectra. (a) Structural similarity index measure; (b) spectral angle mapper; (c) peak signal-to-noise; (d) mean squared error.

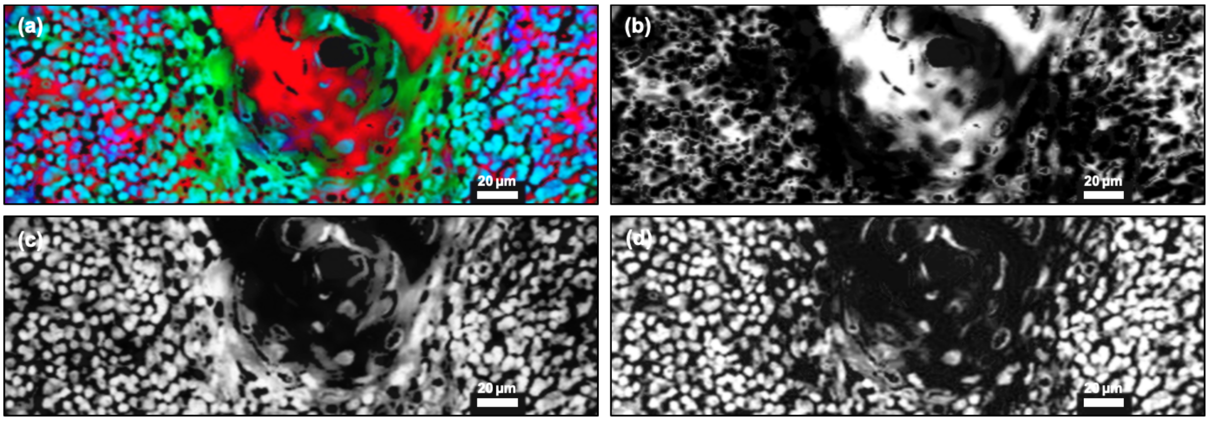

Fig S2: (a) Magnified image of region shown in Fig. 3(b); (b) cytokeratin (red channel); (c) collagen (green channel); (d) ds-DNA (blue channel). In the greyscale images white is high and black is low concentration.

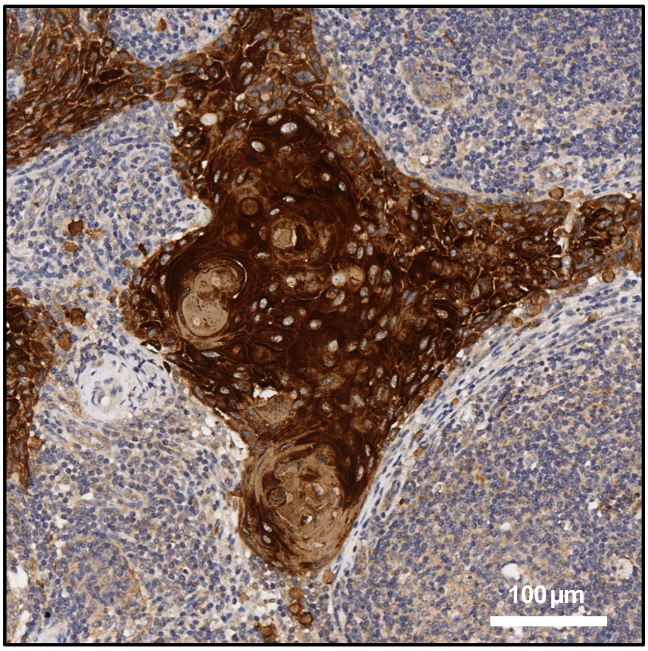

Fig. S3: Cytokeratin stain of the section adjacent to the sections used for the H&E and FTIR images shown in Figs 1-3.

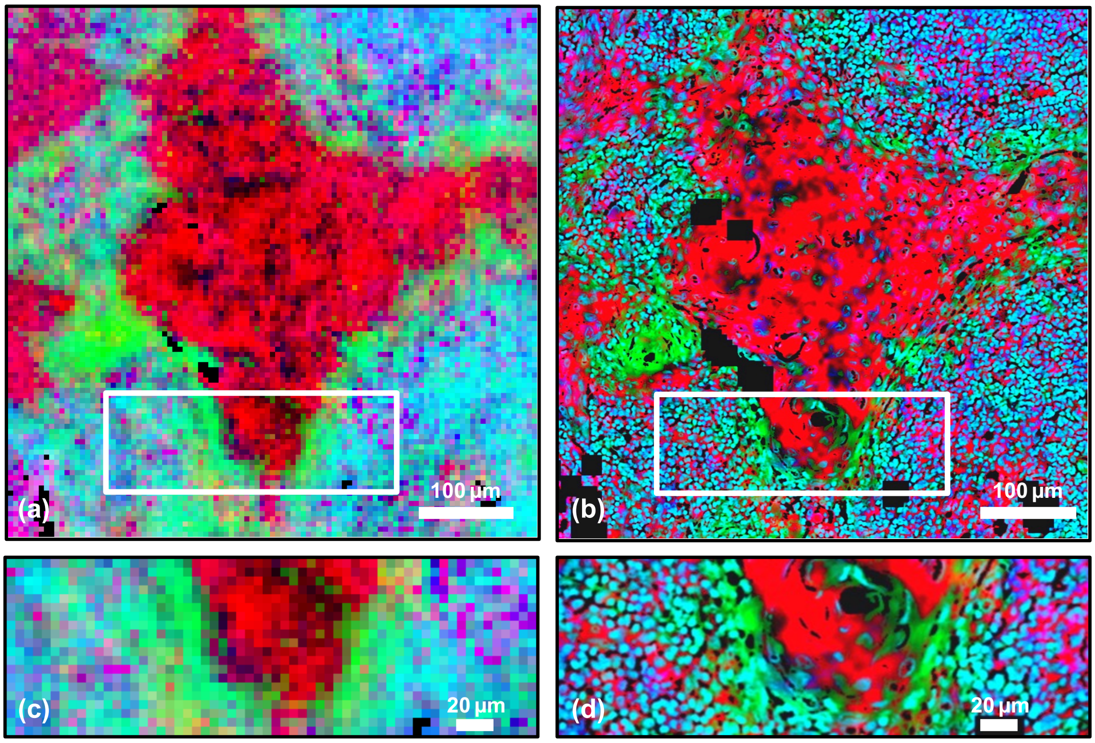

Fig. S4: (a) Least-squares fit of the original (unfused) FTIR spectra displayed as a RGB composite image (red = cytokeratin, green = collagen and blue = ds-DNA); (b) for comparison, Fig. 3(b) is reproduced here. (c) Magnified image taken from the region shown in (d); for comparison, Fig. 3(d) is reproduced.
